# Trends in overweight and obesity in Lebanon: evidence from two national cross-sectional surveys (1997 and 2009)

**DOI:** 10.1186/1471-2458-12-798

**Published:** 2012-09-17

**Authors:** Lara Nasreddine, Farah Naja, Marie Claire Chamieh, Nada Adra, Abla-Mehio Sibai, Nahla Hwalla

**Affiliations:** 1 Department of Nutrition and Food Science, Faculty of Agricultural and Food Sciences, American University of Beirut, Beirut, Lebanon; 2 Department of Epidemiology and Population Health, Faculty of Health Sciences, American University of Beirut, Beirut, Lebanon

**Keywords:** Obesity, Trends, Adults, Children, Adolescents, Lebanon

## Abstract

**Background:**

Even though the obesity epidemic continues to grow in various parts of the world, recent reports have highlighted disparities in obesity trends across countries. There is little empirical evidence on the development and growth of obesity in Lebanon and other countries of the Eastern Mediterranean Region. Acknowledging the need for effective obesity preventive measures and for accurate assessment of trends in the obesity epidemic, this study aims at examining and analyzing secular trends in the prevalence of overweight and obesity over a 12-year period in Lebanon.

**Methods:**

Based on weight and height measurements obtained from two national cross-sectional surveys conducted in 1997 and 2009 on subjects 6 years of age and older, BMI was calculated and the prevalence of obesity was determined based on BMI for adults and BMI z-scores for children and adolescents, according to WHO criteria. Age -and sex- adjusted odds ratios for overweight and obesity were determined, with the 1997 year as the referent category. Annual rates of change in obesity prevalence per sex and age group were also calculated.

**Results:**

The study samples included a total of 2004 subjects in the 1997 survey and 3636 in the 2009 survey. Compared to 1997, mean BMI values were significantly higher in 2009 among all age and sex groups, except for 6–9 year old children. Whereas the prevalence of overweight appeared stable over the study period in both 6–19 year old subjects (20.0% vs. 21.2%) and adults aged 20 years and above (37.0% vs. 36.8%), the prevalence of obesity increased significantly (7.3% vs. 10.9% in 6–19 year olds; 17.4% vs. 28.2% in adults), with the odds of obesity being 2 times higher in 2009 compared to 1997, in both age groups (OR = 1.96, 95% CI:1.29-2.97 and OR = 2.01, 95% CI: 1.67-2.43, respectively). The annual rates of change in obesity prevalence ranged between +4.1% in children and adolescents and +5.2% in adults.

**Conclusion:**

The study’s findings highlight an alarming increase in obesity prevalence in the Lebanese population, over the 12-year study period, and alert to the importance of formulating policies and nutritional strategies to curb the obesity rise in the country.

## Background

The prevalence of obesity is increasing worldwide, reaching alarming levels in several parts of the world, plaguing both high and low-income countries and jeopardizing the ability of these countries to cope with the increasing cost of treatment of obesity associated diseases [1]. The Eastern Mediterranean region is no exception, with obesity rates in its countries reaching high levels, exceeding at times those reported from developed countries such as the USA and Europe [2]. A recent report has highlighted a rapid growth in the prevalence of overweight and obesity in Arabian gulf states including Kuwait, Qatar, Saudi Arabia and Bahrain where 66-75% of the adult population and 25-40% of children and adolescents are currently estimated to be overweight or obese [3].

Even though the obesity epidemic continues to grow in most parts of the world, recent studies have highlighted disparities in obesity trends across countries [4,5]. For example, whereas a tendency towards the stabilization of the obesity epidemic has been noted between 1999 and 2010 in the pediatric population of Australia, Europe and Russia, a decreasing trend in childhood and adolescent obesity was reported from Japan [5]. In contrast, strong increases in obesity prevalence were observed among Chinese and Vietnamese children [5,6]. Similar disparities were observed among adults, with trends indicating a levelling off of the obesity epidemic in the US and some European countries and increasing rates in Canada and several Asian countries [5]. Stemming from this heterogeneity in obesity secular trends, Rokholm and colleagues [5] emphasized the need for detailed studies to monitor obesity trends in different parts of the world in order to identify the driving forces behind the epidemic, set policy priorities and subsequently evaluate their success.

In Lebanon, as in most countries of the Eastern Mediterranean Region, national studies reporting on obesity secular trends are lacking. Lebanon, a small middle-income country with a total population estimated at 3.6 million, is presently experiencing rapid economic, social and cultural changes. Concomitantly, the country is witnessing an accelerated pace of nutrition transition that may be associated with an increased burden of obesity and other non-communicable diseases. A national population-based study conducted in 1997 showed high prevalence rates of obesity among adults (14.5% in men and 18.8% in women), comparable to those observed in developed countries [7]. Available data also suggest that cardiovascular diseases account for around 60% of all-cause mortality in persons aged 50 years and older [8], the prevalence of hypertension increased by 3-fold in the last decade, and the prevalence of the metabolic syndrome in adults was described as notably high (31.2% based on IDF criteria), exceeding that reported from the US, Europe and most neighboring countries [9].

Acknowledging the need for effective obesity preventive measures and for accurate assessment of trends in the obesity epidemic, this study aims at examining and analyzing secular trends in obesity prevalence over a twelve-year period (1997 through 2009) in Lebanon, while focusing on sex-differentials in trends across age strata. The prevalence of obesity was determined according to WHO criteria based on BMI for adults and BMI z-scores for children and adolescents [10,11]. For the pediatric population, the IOTF and CDC criteria [12,13] were also used to allow for comparison with regional and international studies. The authors hypothesize that the prevalence of obesity has significantly increased over the 12 year study period in Lebanon, as the nutrition transition is unfolding in the country with accelerated rates of modernization, urbanization, cultural and lifestyle changes.

## Methods

### Study design and subjects

The data for the present analysis were obtained from two national cross-sectional surveys conducted in Lebanon in two time periods, 1997 and 2009. The two studies were executed by the same investigators (AMS and NH) at the American University of Beirut, and similar procedures and data collection methods were used in both surveys. Both the 1997 and 2009 study samples were based on sampling frames provided by nationally representative household-based surveys conducted by the Ministry of Social Affairs [14,15], and in both studies, recruitment efforts targeted a sample with an age, sex and district distribution proportionate to that of the Lebanese population [14,15].

The design and conduct of the 1997 study has been described in detail elsewhere [7]. Briefly, the study sample was based on the sampling frame provided by the Lebanese Ministry of Social Affairs in collaboration with UNFPA according to the Population and Housing Survey (PHS) [15], which was a nationally representative survey of households in each of the country’s six governorates and 26 districts. A random sub-sample of 1% of the PHS household sampling frame was selected for the 1997 study.

The 2009 study sample was based on the sampling frame provided by the Ministry of Social Affairs/Central Administration of Statistics in collaboration with UNDP, according to the National Survey of Household Living Conditions [14], which covered primary residences across the Lebanese territory. Sample size calculation for the 2009 study was performed based on previously estimated prevalence rates for the main outcome of interest. As such, the prevalence rate of obesity (17% among adults [7]) was taken as the reference within an allowed error of 1.5% and 95% confidence.

For both the 1997 and 2009 surveys, study samples were drawn from randomly selected households, based on stratified cluster sampling: the strata were the Lebanese Governorates, the clusters were selected further at the level of districts, urban and rural areas, and the housing units constituted the primary sampling units in the different districts of Lebanon. One adult from each household and one child/adolescent from every other household were selected from the household roster, excluding pregnant and lactating women and subjects with mental disabilities. For the 1997 survey, field work was conducted between April 1997 and September 1997, and the final sample consisted of a total of 2004 subjects, while for the 2009 study, field work was carried between May 2008 and August 2009 with a final sample including 3636 subjects;

The design and conduct of both surveys were approved by the Institutional Review Board of the American University of Beirut, and informed consent from adults/parents and informed assent from children and adolescents were obtained prior to enrollment in the studies.

### Interview schedules and anthropometric measurements

Subjects were interviewed in the household setting. Face-to-face interviews were conducted using a multicomponent questionnaire covering information on demographic, socioeconomic and lifestyle characteristics in addition to medical history and health seeking behavior. Among other variables, the questionnaire provided information on sex, age, family history of obesity, education, sitting time and crowding index. Interviews were conducted by trained nutritionists and lasted for approximately one hour.

Anthropometric measurements were taken using standardized protocols [16] and calibrated equipment. Height and body weight were measured according to standard procedures, using a portable stadiometer (Holtain, Crymych, UK) and a SECA-calibrated electronic weighing scale (Hamburg, Germany), respectively. Subjects were weighed to the nearest 0.1 kg in light indoor clothing and with bare feet or stockings. Height was measured without shoes and recorded to the nearest 0.5 cm. Anthropometric measurements were taken and recorded by trained nutritionists who were working in teams of two, the examiner and the recorder. All measurements were taken twice and the average of the 2 values adopted. Body mass index (BMI) was calculated as the ratio of weight (kilograms) to the square of height (meters).

### Definitions of overweight and obesity

For children and adolescents, overweight and obesity were defined based on sex and age-specific +1 and +2 BMI z-scores, respectively, according to the WHO new growth standards [11]. WHO software (Anthro Plus for PC) was used to calculate BMI z-score for each specific age and sex. For comparative purposes with other studies conducted worldwide, prevalence rates of overweight and obesity were also determined using the International Obesity Taskforce (IOTF) [12] and the CDC 2000 criteria [13].

For adults aged 20 years or older, overweight was defined as a BMI of 25.0 to 29.9 and obesity was defined as a BMI of 30.0 or higher [10].

### Statistical analysis

Descriptive statistics were calculated for BMI, and results were expressed as means, SDs and percentiles. Normality of BMI was confirmed and differences in mean BMIs between 1997 and 2009 were examined using the independent student *t*-test. Differences in obesity and overweight prevalence rates between the two survey years were examined using chi-square test. Annual rates of change in obesity prevalence per sex and age group were calculated. Multivariate logistic regression was also conducted to calculate the odds of obesity and overweight should the individual belong to the 2009 survey as compared to the 1997 survey. In this model, survey year was the independent variable, with year 1997 used as the referent category, and with obesity or overweight as the dependant variable. The regression model was adjusted for age (linear variable), sex, family history of obesity (yes/no), father and mother education (high/low), crowding index (< 1 person/room/≥1 person/room) and marital status (married/not married). To account for sampling effect, weighted analyses were conducted. In weights, the population distribution by sex and age, as determined by the Central Administration of Statistics [14,15] was used as the reference.

The Statistical Package for the Social Sciences (SPSS, version 18) and the STATA software (STATA, release 11) were used and a *p*-value <0.05 was considered significant.

## Results

The age and sex distributions of both study samples are shown in Table 1. Refusal rates at the household level were similar between both surveys (10% in 1997 and 10.7% in 2009), yielding a total of 2004 subjects in the 1997 survey and 3636 in the 2009 survey. The main reasons for refusing to participate in the survey included lack of time or disinterest in the study.

**Table 1:** Distribution of the study samples by age, sex and survey year

**Age groups (years)**	**1997**			**2009**		
	**Males**	**Females**	**Both Sexes**	**Males**	**Females**	**Both Sexes**
	**n = 859**	**n = 1145**	**n = 2004**	**n = 1720**	**n = 1916**	**n = 3636**
		**n(%)**			**n(%)**	
6-9	102(11.9)	94(8.2)	196(9.8)	131(7.6)	119(6.2)	250(6.9)
10-19	256(29.8)	336(29.3)	592(29.5)	345(20.1)	344(18.0)	689(18.9)
20-39	198(23.1)	370(32.3)	568(28.3)	649(37.7)	785(41.0)	1434(39.4)
40-59	183(21.3)	243(21.2)	426(21.3)	373(21.7)	449(23.4)	822(22.6)
≥60	120(14.0)	102(8.9)	222(11.1)	222(12.9)	219(11.4)	441(12.1)

**Figure 1:**
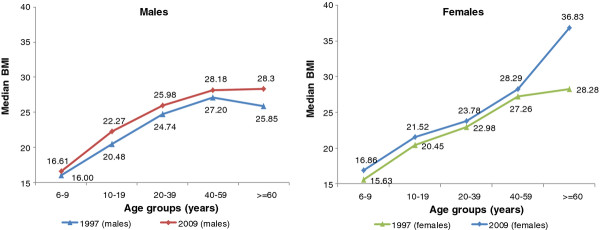
Median body mass index of study subjects by sex, age group and survey year in the study population, Lebanon 1997-2009.

Means of BMI, weight and height (±SD) as stratified by age, sex and survey year are presented in Table 2 for 6–19 year old subjects and in Table 3 for adults aged 20 years and above. Tables 2 and 3 also display secular trends in BMI percentiles for the two study periods, with trends in the 50^th^ BMI percentiles being also shown in Figure 1. There were significant increases in height in both the pediatric and adult populations, with the increases over the 12 year study period being considerably higher in children and adolescents (+16.39 cm in boys and +12.33 cm in girls) as compared to adults (+1.65 cm in men and + 0.62 cm in women). Significant increases in the population’s weight were also observed with the increases being higher in the pediatric population (+19 kg in boys and +12.91 kg in girls) as compared to adults (+6.71 kg in men and + 3.86 kg in women). Findings also show that mean BMI values were significantly higher in 2009 as compared to 1997 in all age and sex groups, except for 6–9 year old children. Amongst 6–19 years old subjects, the BMI 85^th^ percentile values increased by 3.65 kg/m^2^ in boys and 2.55 kg/m^2^ in girls, with similar increases observed in the 95^th^ percentile values which amounted to 3.77 kg/m^2^ in boys and 2.63 kg/m^2^ in girls. Among adults older than 20 years, the BMI 95^th^ percentile increased by 2.52 kg/m^2^ in men and 3.69 kg/m^2^ in women.

**Table 2:** Anthropometric measurements by age, sex and survey year in the study population aged 6–19 years, Lebanon 1997-2009

	**Mean ± SD**			
	**Males**		**Females**	
	**1997**	**2009**	**1997**	**2009**
**Ages 6–19 years**				
** 6-9 yrs**				
Weight, mean (Kg)	25.87 ± 6.63	29.61 ± 9.51	25.73 ± 7.86	28.85 ± 7.76
Height, mean (cm)	124.52 ± 9.33	128.44 ± 10.43	124.13 ± 10.40	127.50 ± 10.10
BMI, mean (kg/m^2^**)**	16.48 ± 2.55	17.58 ± 3.45	16.51 ± 3.78	17.52 ± 2.90
5^th^ percentile	13.71	13.87	13.14	13.90
10^th^ percentile	14.08	14.29	13.98	14.61
85^th^ percentile	20.59	21.02	20.12	20.79
95^th^ percentile	--	24.61	--	23.26
** 10-19 yrs**				
Weight, mean (Kg)	52.39 ± 17.98^a^	68.54 ± 18.17^a^	50.14 ± 11.56^b^	57.23 ± 12.17^b^
Height, mean (cm)	157.49 ± 16.14^a^	169.49 ± 11.25^a^	155.72 ± 10.06^b^	160.14 ± 8.45^b^
BMI, mean (kg/m^2^**)**	20.51 ± 4.28^a^	23.49 ± 4.94^a^	20.47 ± 3.49^b^	22.19 ± 4.02^b^
5^th^ percentile	14.91	17.31	15.55	17.23
10^th^ percentile	15.54	18.00	16.23	17.68
85^th^ percentile	25.25	28.50	23.92	25.98
95^th^ percentile	31.81	33.61	27.33	29.85
** Total**				
Weight, mean (Kg)	44.84 ± 19.68^a^	63.84 ± 21.50^a^	41.38 ± 15.68^b^	54.29 ± 14.63^b^
Height, mean (cm)	148.10 ± 20.85^a^	164.49 ± 17.45^a^	144.39 ± 18.32^b^	156.72 ± 13.21^b^
BMI, mean (kg/m^2^**)**	19.36 ± 4.25^a^	22.77 ± 5.16^a^	19.05 ± 4.04^b^	21.70 ± 4.17^b^
5^th^ percentile	14.24	15.65	14.05	15.80
10^th^ percentile	14.92	16.69	14.61	17.22
85^th^ percentile	24.09	27.74	23.23	25.78
95^th^ percentile	29.06	32.83	26.48	29.11

^ab^ Mean values with the same superscripts are significantly different between the two surveys at p < 0.05.

**Table 3:** Anthropometric measurements by age, sex and survey year in the study population aged ≥20 years, Lebanon 1997-2009

	**Mean ± SD**			
	**Males**		**Females**	
	**1997**	**2009**	**1997**	**2009**
**Age ≥ 20 years**				
** 20-39 yrs**				
Weight, mean (Kg)	76.08±13.63^a^	81.99±16.21^a^	61.81±10.87^b^	64.17±12.69^b^
Height, mean (cm)	173.93±7.58^a^	175.27±6.77^a^	160.83±6.37	161.34±5.93
BMI, mean (kg/m^2^**)**	25.09±4.00^a^	26.63±4.71^a^	23.89±3.98^b^	24.68±4.83^b^
5^th^ percentile	18.68	20.20	18.66	18.76
10^th^ percentile	20.06	21.22	19.53	19.40
85^th^ percentile	29.08	31.15	27.96	29.80
95^th^ percentile	32.92	35.01	31.83	34.16
** 40-59 yrs**				
Weight, mean (Kg)	77.42±12.08^a^	85.52±15.20^a^	68.72±12.27^b^	72.82±15.09^b^
Height, mean (cm)	169.22±6.34^a^	172.65±6.95^a^	157.02±7.09^b^	158.13±5.79^b^
BMI, mean (kg/m^2^**)**	26.98±3.86^a^	28.68±4.81^a^	27.88±4.86^b^	29.16±6.05^b^
5^th^ percentile	20.56	21.44	21.20	20.89
10^th^ percentile	22.09	23.08	22.16	22.20
85^th^ percentile	30.63	33.00	32.87	35.41
95^th^ percentile	34.15	36.96	36.27	40.63
** ≥60 yrs**				
Weight, mean (Kg)	72.06±14.28^a^	78.88±14.09^a^	66.79±13.13^b^	71.68±14.42^b^
Height, mean (cm)	166.34±7.4	167.38±6.91	152.17±6.00^b^	154.16±6.49^b^
BMI, mean (kg/m^2^**)**	25.94±4.27^a^	28.02±4.26^a^	28.84±5.49^b^	30.20±6.07^b^
5^th^ percentile	18.89	20.31	21.88	21.52
10^th^ percentile	20.93	22.53	22.38	23.20
85^th^ percentile	30.03	32.41	32.90	36.83
95^th^ percentile	33.06	34.16	38.06	40.87
** Total**				
Weight, mean (Kg)	75.79±13.42^a^	82.50±15.66^a^	64.62±12.09^b^	68.48±14.45^b^
Height, mean (cm)	171.30±7.82^a^	172.95±7.44^a^	158.29±7.27^b^	158.91±6.5^b^
BMI, mean (kg/m^2^**)**	25.7±4.09^a^	27.54±4.75^a^	25.86±5.02^b^	27.22±6.02^b^
5^th^ percentile	19.14	20.41	19.10	19.26
10^th^ percentile	20.46	21.84	20.14	20.26
85^th^ percentile	29.84	31.96	31.22	33.49
95^th^ percentile	33.06	35.58	34.85	38.54

^ab^ Mean values with the same superscripts are significantly different between the two surveys at p<0.05.

Table 4 shows the prevalence estimates of overweight and obesity among children and adolescents aged 6 to 19 years by sex, age group and survey year. Overall, obesity rates were almost double in boys as compared to girls in both the 1997 survey (10.2% and 5.4%, respectively) and the 2009 study (15.5% and 6.9%, respectively), with higher sex-based differentials being evident among adolescents. Whereas the prevalence of overweight appeared relatively stable between 1997 and 2009 in 6–19 year old subjects (20.0% and 21.2%, respectively), obesity was found to increase significantly during this period (7.3% and 10.9%). The annual rates of change were higher among 6–9 year old boys compared to girls (+6% vs. +2.6%), while among 10–19 year olds, the relative increase was considerably higher among adolescent girls compared to boys (+8.9% vs. +4.5%).

**Table 4:** Trends in overweight and obesity prevalence by sex and age group for the study population, Lebanon 1997- 2009

**Children and Adolescents (6–19 years)**									
**Overweight only (+1 < BMI z-score ≤ +2)**				**Obese (BMI z-score > +2)**			**Overweight & Obese (BMI z-score > +1)**		
	**1997%**	**2009%**	**% Av. Annual change**	**1997%**	**2009%**	**% Av. Annual change**	**1997%**	**2009%**	**% Av. Annual change**
**Children (6–9 years)**									
Boys^†^	16.5	17.2	+0.4	11.3	19.5	+6.0	27.8	36.7	+2.7
Girls^†^	15.2	23.9	+4.8	9.8	12.8	+2.6	25.0	36.7	+3.9
Both sexes^††^	15.7	20.6	+2.6	10.3	16.1	4.7	26.0^a^	36.7 ^a^	+3.4
**Adolescents (10–19 years)**									
Boys^†^	24.3	23.8	−0.2	9.7	14.9	+4.5	34.0	38.7	+1.2
Girls^†^	20.5	19.2	−0.5	3.0	6.2	+8.9	23.5	25.4	+0.7
Both sexes^††^	22.1	21.3	−0.3	5.9^a^	10.2 ^a^	6.1	28.0	31.5	+1.0
**Total**									
Boys^†^	22.1	23.0	+0.3	10.2^a^	15.5 ^a^	+4.3	32.3	38.5	+1.6
Girls^†^	18.6	19.7	+0.5	5.4	6.9	+2.3	24.0	26.6	+0.9
Both sexes^††^	20.0	21.2	+0.5	7.3^a^	10.9 ^a^	+4.1	27.3	32.1	+1.5
**Adults (≥20 years)**									
**Adults (20–39 years)**									
Men^†^	39.5	40.3	+0.2	11.9^a^	20.4 ^a^	+6.0	51.4^a^	60.7 ^a^	+1.5
Women^†^	23.3	24.9	+0.6	8.1^a^	14.8 ^a^	+6.9	31.4^a^	39.7 ^a^	+2.2
Both sexes^††^	30.2	32.0	+0.5	9.8 ^a^	17.4 ^a^	+6.5	40.0^a^	49.4 ^a^	+2.0
**Adults (40–59 years)**									
Men^†^	52.8	45.8	−1.1	19.8^a^	34.7 ^a^	+6.3	72.6^a^	80.5 ^a^	+0.9
Women^†^	44.0	40.3	−0.7	28.7^a^	36.6 ^a^	+2.3	72.7	76.9	+0.5
Both sexes^††^	47.8	42.7	−0.9	24.8^a^	35.7 ^a^	+3.7	72.6^a^	78.4 ^a^	+0.7
**Adults (≥60 years)**									
Men^†^	45.7	43.2	−0.5	15.5^a^	33.4 ^a^	+9.6	61.2^a^	76.6 ^a^	+2.1
Women^†^	35.7	35.1	−0.1	38.4	49.3	+2.4	74.1^a^	84.4 ^a^	+1.2
Both sexes^††^	39.9	38.8	−0.2	28.7^a^	42.1 ^a^	+3.9	68.6^a^	80.9 ^a^	+1.5
**Total**									
Men^†^	44.4	42.6	−0.3	14.8^a^	27.4 ^a^	+7.1	59.2^a^	70.0 ^a^	+1.5
Women^†^	31.4	32.1	+0.2	19.3^a^	28.8 ^a^	+4.1	50.7^a^	60.9 ^a^	+1.7
Both sexes^††^	37.0	36.8	0.0	17.4^a^	28.2 ^a^	+5.2	54.4^a^	65.0 ^a^	+1.6

^a^ Values with the same superscripts are significantly different between the two surveys at p < 0.05.

Findings for adults aged 20 years and above are also presented in Table 4. In both the 1997 and 2009 surveys, obesity was higher among men compared to women in the younger age groups (20–39 years) while in the older age groups (40–59 years and ≥ 60 years), the prevalence of obesity was higher in women compared to men. Overall, the prevalence of overweight appeared relatively stable between 1997 and 2009 (37.0% and 36.8%, respectively), whereas the prevalence of obesity was found to increase significantly between the two time periods (17.4% and 28.2%, respectively); with the increase being consistent across age groups. The highest relative increases in obesity prevalence over time were documented among men (+7.1%) compared to women (+4.1%), except for 20–39 year old adults.

Logistic regression analyses indicated a statistically significant increase in obesity risk among children and adolescents, with the odds increasing by two-fold in the 2009 survey as compared to 1997 (OR = 1.96, 95% CI: 1.29-2.97) among 6–19 year olds (data not shown). A similar increase in obesity risk was noted among all age and sex groups in the adult population with the odds of obesity being 2 times higher in 2009 compared to 1997 in subjects aged 20 years and above (OR = 2.01, 95% CI: 1.67-2.43) (data not shown).

**Figure 2:**
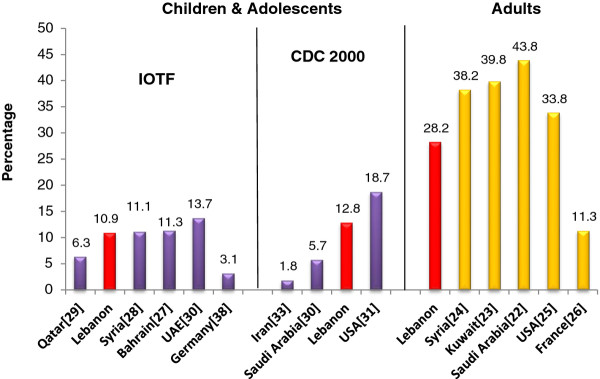
**Prevalence (%) of obesity among Lebanese adults, children and adolescents (using both IOTF and CDC criteria**[12,13]**) compared to those reported from selected countries of the Eastern Mediterranean region & worldwide.**

Figure 2 reproduces available data for obesity in selected countries of the region and worldwide and compares them with those obtained in this study. To allow comparisons with findings reported from other countries, the prevalence of obesity among children and adolescents was re-calculated according to the IOTF and CDC 2000 criteria.

## Discussion

Studies investigating secular changes in the prevalence of overweight and obesity and in populations’ anthropometric measurements are scarce in the Eastern Mediterranean region. The present paper is the first to report on overweight and obesity trends in the Lebanese population and constitutes an eye opener for other countries of the region. Importantly, the study documented a rapid increase in BMI across sex and age groups and all percentile strata between 1997 and 2009, suggesting that the population’s BMI distribution is shifting to the right. It also showed that, although both weight and height of children, adolescents and adults have significantly increased in Lebanon over the 12 year study period, the relative changes in mean weight (+42.4% in boys; +31.2% in girls; +8.9% in men; +5.97% in women) have by far surpassed changes in mean height (+11% in boys; +8.5% in girls; 0.96% in men; 0.39% in women), which explains the observed significant increases in mean BMI across sex and age groups. In adults, the increase in mean BMI over the 12 year study period ranged between 1.36 kg/m^2^ in women and 1.84 kg/m^2^ in men, thus exceeding the recently reported worldwide estimate (0.5 kg/m^2^ per decade for women and 0.4 kg/m^2^ per decade for men), and surpassing BMI increases in high income countries such as the USA (1.2 kg/m^2^ per decade for women 1.1 kg/m^2^ per decade for men) [4]. The study has also documented a consistent increase in the 85^th^ and 95^th^ percentile BMI values across various sex and age groups, with the increase reaching as high as 4 kg/m^2^ in some population groups, notably 6–19 year old boys and adult women. These findings carry public health implications, given that the association between BMI and chronic diseases is continuous and entails a dose–response relationship [4,17]. A collaborative analysis of data from almost 900 000 adults in 57 prospective studies indicated that each 5 kg/m^2^ increase in BMI above the optimal range of 22.5–25 kg/m^2^ was associated with a 30% increase in all-cause mortality (40% for vascular; 60–120% for diabetic, renal, and hepatic; 10% for neoplastic; and 20% for respiratory and for all other mortality [17].

Parallel to the observed shift in the population’s BMI, the findings of this study highlight, as hypothesized, an escalating rate of obesity among both the adult and pediatric populations, whereas the prevalence of overweight appeared stable over the study period. The observed increasing trend in adult obesity (17.4% vs. 28.2%) is not limited to Lebanon. Iran, a neighboring country in the region, is experiencing similar increases in adult obesity which has escalated from 13.6% in 1999 to 22.3% in 2007 [18]. Reports from Arabian Gulf countries have also suggested increases in adult obesity rates, with the highest increase reported from Saudi-Arabia and Kuwait [3]. Based on a recent review by Ng et al. (2010), the prevalence of obesity amongst 30–60 year old Saudi adults has risen from 36 to 50% among women and from 23 to 32% among men between 1993 and 2005. In parallel, obesity among Kuwaiti adults was reported to increase from 48 to 58% in women and from 31 to 46% in men [3]. Noteworthy is the fact that the annual rates of increase in obesity prevalence among Lebanese adults (+4.1% in Lebanese women and +7.1% in men) are higher than those reported from Arabian Gulf states, where the annual rates of change did not exceed +1.7% in women and +4.1% in men. Increasing obesity trends among adults have been also reported from other developing Asian countries, namely India, Nepal, Bangladesh and Malaysia, whereas governmental efforts to address obesity have resulted in the stabilization or levelling off of the obesity epidemic among adults in other parts of the world, including France, England and the US [5].

This study has also importantly documented a significant increase in the prevalence of obesity among Lebanese youth (7.3% vs. 10.9%), with the odds of obesity being approximately 2 times higher in 2009 as compared to 1997 in 6–19 year old children and adolescents. Data on obesity trends in children and adolescents from the Eastern Mediterranean region (EMRO) are scarce. Based on a review article by Wang and Lobstein (2006), obesity rates among school age children in the EMRO region was estimated at 9.4% in 2006 and was projected to increase to 11.5% in 2010, with an annual increase estimated at +5.6%, a value that is slightly higher than the annual rate of change documented among Lebanese children and adolescents in this study (+4.1%) [19]. The rise in childhood obesity in Lebanon is parallel to the rise witnessed by other countries in transition, such as China and Vietnam, whereas evidence for stability or levelling off of the obesity epidemic was reported among children and adolescents from Australia, England, France, Greece, Sweden and the USA. Strong evidence suggests that childhood obesity rates appear linked to socio-economic development, changes in environmental factors such as living and school environments and physical activity patterns [19]. Further analyses of data provided by the 1997 and 2009 national surveys showed that sedentary behavior among Lebanese children and adolescents (defined as ≥ 10 h sitting time per day) increased from 19.9% in 1997 to 60.5% in 2009, a finding that may mirror the increased reliance of youth on satellite TV, computers and computer games, as well as telecommunication technology. Concomitantly, while maternal education did not show considerable changes over time, secular shifts in father education levels were observed with a significant decrease in the proportion of fathers who have completed University education (25.7% in 1997 vs. 16.3% in 2009). Paternal education has been suggested as one of the factors modulating obesity risk in childhood [20]. A study conducted in Italy among 8- to 9-year-old children [20] showed that the prevalence of pediatric obesity was inversely related to the educational level of fathers, thus highlighting the role of paternal education in modulating the family’s lifestyle, economic and cultural resources, all of which may bear ramifications on nutritional and behavioral choices.

In this study, sex-based differentials in obesity trends were observed. Even though obesity prevalence rates were higher in adult women (>20 years old) compared to men in both surveys, the highest relative increases in obesity prevalence over time were documented among men (+7.1% in men compared to +4.1% in women). These findings are in agreement with those reported by Ng et al. (2010) among 30–60 year old adults from Saudi Arabia and Bahrain where annual rates of change in obesity prevalence were consistently higher among men compared to women [3]. Similarly, a recent study conducted in Kuwait illustrated a gain of 1.78 kg/m^2^ in mean BMI among adult men between 2001and 2005, with smaller gains documented among women (1.16 kg/m^2^) [21]. Sex-based differentials were also noted in the pediatric population, with the relative increases in obesity prevalence being higher among 6–9 year old boys compared to girls (+6% vs. +2.6%), while among adolescents, the relative increase was almost double in girls as compared to boys (+8.9% vs. +4.5%). These findings are in agreement with reports from Bahrain where the prevalence of obesity was found to escalate over time among adolescents girls (from 17.9% in 2000 to 19.4% in 2008) but not among boys. Differences in secular trends by sex point towards socio-cultural and biological factors that are important to elucidate and highlight the need for reliable longitudinal studies to follow children over time as they move from infancy to adolescence to track growth and the incidence of obesity over time.

At present, and based on the 2009 survey data, obesity prevalence among Lebanese adults aged 20 years and over (27.4% in men and 28.8% in women) is still lower than estimates reported from several Eastern Mediterranean countries such as Saudi Arabia (43.8%) [22] Kuwait (39.8%) [23] Syria (38.2%) [24] and the US (33.8%) [25], while exceeding the reported age-standardized prevalence of obesity worldwide (9.8% in men and 13.8% in women, in 2008) and surpassing obesity rates reported from several countries in Western and Northern Europe where estimates of obesity prevalence range between 9.7 and 14.7% [26]. The high prevalence of obesity in the Lebanese adult population, coupled with the observed increasing secular trend in its prevalence, underlines obesity as a rapidly progressing pandemic in the country.

Using the IOTF criteria and based on the 2009 survey, current obesity rates amongst 6–19 years old children and adolescents in Lebanon (10.9%) appear to be comparable to estimates from Bahrain (11.3%) [27] and Syria (11.1%) [28], higher than those observed in Qatar (6.3%) [29], while being lower than those reported from the UAE (13.7%) [30]. Using the CDC 2000 criteria, the prevalence of obesity in Lebanese children and adolescents (12.8%) appears lower than that reported from the US (18.7%) [31], while being considerably higher than obesity rates reported from Iran (1.8%) [32] and Saudi-Arabia (5.7%) [33]. The observed growing obesity epidemic in this age group is of major public health concern. Pediatric obesity has been shown to track into adulthood and to predict a broad range of adverse health effects [6]. With those below 20 years making up close to 50% of the Lebanese population [34], these estimates do not bode well for the health and well-being of the population.

Little is known about the underlying causes of the increasing trends in obesity prevalence in Lebanon. In general, it appears that a permissive environment with few constraints on food intake and reduced physical activity, in combination with evolutionary forces adapted to a drastically different environment, can lead to overweight and obesity in many individuals [35]. It is thus probable that shifts in the Lebanese population’s food consumption patterns and lifestyle may have contributed to the alarming increase in obesity rates. Like other countries of the Eastern Mediterranean Region, Lebanon is now facing a fast rate of development and urbanization, with 90% of the Lebanese population being currently classified as urban [34]. This is accompanied by a concomitant nutrition transition characterized by changes in food supply and intake in addition to reduced physical activity. Trends in dietary patterns in Lebanon illustrate a consistent rise in total energy supply, amounting to 850 kcal per capita per day between 1970 and 2005 [2]. Physical inactivity was also found to be highly prevalent in Lebanon with 68.7% of the Lebanese adult population estimated to be physically inactive [2]. Fazah et al. [36] have recently showed that, amongst Lebanese adolescents (14–18 years old), total screen time including television viewing, computer and videogames, ranged between 26.5 and 33 h/week thus exceeding values reported for US adolescents, which ranged between 15.3 and 21.2 h/week [37].

The increases in obesity prevalence raise questions about its implications for health and disease burden in Lebanon. Of more concern is the observed increasing trend in obesity among Lebanese children and adolescents, given its influence on young people’s psychosocial development and its associated health consequences [38]. A recent study indicated that the prevalence of the metabolic syndrome among prepubertal obese Lebanese children (31.9%) is considerably high, surpassing that reported among obese children from several parts of the world [2].

The results of this study should be considered in light of the following limitations. Obesity prevalence rates and trends were based on two distinct points in time only and were obtained from sample surveys, which like other survey data, may be subject to sampling error or non-sampling error. In addition the prevalence of obesity was based on BMI, which although widely used as a surrogate measure of adiposity, is in fact a measure of excess weight relative to height, rather than a measure of excess body fat or fat distribution.

## Conclusion

This study showed that the prevalence of obesity increased significantly, by two-fold over the 12-year study period in both the pediatric and the adult populations of Lebanon. More alarming are the annual rates of increase in obesity prevalence, which appear to be higher than those reported from other countries in the EMRO region. Findings of the present study should alert to the importance of formulating policies and strategies to curb the progression of obesity in Lebanon. Population-wide community-based intervention programs that involve multisectoral partnerships and that are responsive to the sociocultural norms of the population must be put in place to hamper the growth of obesity and associated non-communicable diseases in Lebanon. This study should be viewed as a basis for future investigations aiming at providing additional data on obesity secular trends in the country to support and confirm the findings of the present study.

## Competing interests

The authors declare that they have no competing interests.

## Authors’ contributions

LN and FN wrote the paper; NH and AMS designed and supervised the study and critically reviewed the paper; MCC participated in data collection for adult subjects as part of the 2009 survey; LN, FN and NA performed data analysis and interpretation; LN and FN contributed equally to this manuscript. All authors contributed to writing the paper and discussed the results. All authors read and approved the final manuscript.

## Authors’ information

All authors are members of the Public Health and Nutrition (PHAN) Research Group at the American University of Beirut, Lebanon.

## Pre-publication history

The pre-publication history for this paper can be accessed here:

http://www.biomedcentral.com/1471-2458/12/798/prepub
